# Thermo-Mechanical Behavior and Strain Rate Sensitivity of 3D-Printed Polylactic Acid (PLA) below Glass Transition Temperature (T_g_)

**DOI:** 10.3390/polym16111526

**Published:** 2024-05-29

**Authors:** Vukašin Slavković, Blaž Hanželič, Vasja Plesec, Strahinja Milenković, Gregor Harih

**Affiliations:** 1 Faculty of Engineering, University of Kragujevac, Sestre Janjic 6, 34000 Kragujevac, Serbia; strahinja.milenkovic@fink.rs; 2 Laboratory for Integrated Product Development and CAD, Faculty of Mechanical Engineering, University of Maribor, Smetanova ulica 17, SI-2000 Maribor, Slovenia; blaz.hanzelic1@um.si (B.H.); vasja.plesec@um.si (V.P.); gregor.harih@um.si (G.H.)

**Keywords:** smart materials, shape memory polymer, 3D printing, 4D printing, thermo-mechanical experiments

## Abstract

This study investigated the thermomechanical behavior of 4D-printed polylactic acid (PLA), focusing on its response to varying temperatures and strain rates in a wide range below the glass transition temperature (T_g_). The material was characterized using tension, compression, and dynamic mechanical thermal analysis (DMTA), confirming PLA’s strong dependency on strain rate and temperature. The glass transition temperature of 4D-printed PLA was determined to be 65 °C using a thermal analysis (DMTA). The elastic modulus changed from 1045.7 MPa in the glassy phase to 1.2 MPa in the rubber phase, showing the great shape memory potential of 4D-printed PLA. The filament tension tests revealed that the material’s yield stress strongly depended on the strain rate at room temperature, with values ranging from 56 MPa to 43 MPA as the strain rate decreased. Using a commercial FDM Ultimaker printer, cylindrical compression samples were 3D-printed and then characterized under thermo-mechanical conditions. Thermo-mechanical compression tests were conducted at strain rates ranging from 0.0001 s^−1^ to 0.1 s^−1^ and at temperatures below the glass transition temperature (T_g_) at 25, 37, and 50 °C. The conducted experimental tests showed that the material had distinct yield stress, strain softening, and strain hardening at very large deformations. Clear strain rate dependence was observed, particularly at quasi-static rates, with the temperature and strain rate significantly influencing PLA’s mechanical properties, including yield stress. Yield stress values varied from 110 MPa at room temperature with a strain rate of 0.1 s^−1^ to 42 MPa at 50 °C with a strain rate of 0.0001 s^−1^. This study also included thermo-mechanical adiabatic tests, which revealed that higher strain rates of 0.01 s^−1^ and 0.1 s^−1^ led to self-heating due to non-dissipated generated heat. This internal heating caused additional softening at higher strain rates and lower stress values. Thermal imaging revealed temperature increases of 15 °C and 18 °C for strain rates of 0.01 s^−1^ and 0.1 s^−1^, respectively.

## 1. Introduction

Smart materials are a prominent class of materials that have revolutionized both research and engineering. In general, materials with shape memory, usually named shape memory materials (SMMs), are characterized by the shape memory effect (SME). SMMs are divided into several groups: shape memory polymers (SMPs), shape memory alloys (SMAs), shape memory hydrogels (SMHs), and shape memory ceramics (SMCs). SMPs can respond to a various external stimulus and can recover their deformed shape and return to their permanent shape from a programmed (temporary) shape under the influence of light [1,2], heat [3], magnetic [4], electricity [5], moisture [6], and water [7,8]. For a long time, SMAs have been very prevalent, especially in human medicine [9], aerospace [10], and robotics [11]. However, today, SMPs and SMHs are slowly taking the lead among other SMMs due to their broad applicability and the relatively low cost of the raw material and manufacturing. The advantages of SMPs and SMHs over, primarily SMAs, are that the stiffness can be adjusted in a wide glass transition temperature range T_g_ (55–100 °C) [12]. Besides that, SMPs are characterized by low density (≈1.2 g/cm^3^), large deformations, biodegradability, biocompatibility, as well as low thermal conductivity [13,14]. SMPs can also restore shape after being exposed to very large plastic deformations of ≈500%, while for SMAs, it is ≈6–7%. In addition to mechanical factors, technological factors such as cost, fabrication, toxicity, or recycling potential significantly affect the predominance of SMPs over SMAs [15] in the era of green technologies and green polymers [16].

**Figure 1:**
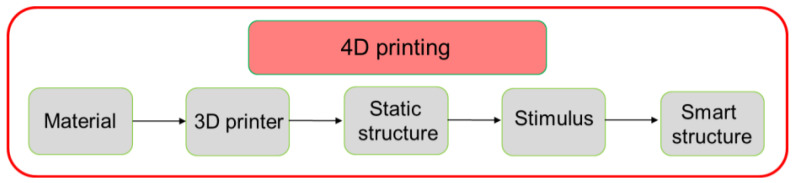
The 4D printing concept with PLA material.

The emergence of 4D printing represents an innovative fusion of smart materials and additive manufacturing techniques, propelling scientific exploration into material responsiveness to external stimuli and the development of intelligent structures for various applications. Smart materials in 4D printing adapt their properties or shapes in response to external stimuli. These materials can also harness energy, typically thermal, to perform mechanical tasks [17]. Four-dimensional printing technologies have facilitated scientific exploration into material research, stimulus responsiveness, mathematical modeling, and the subsequent development of intelligent structures. Four-dimensional printing has garnered increased interest lately, notably through the pioneering work of Professor Tibbits’ research group at the Massachusetts Institute of Technology (MIT) [18]. Like most rapidly growing technologies, 4D printing relies on the rapid development of smart materials, 3D printers, mathematical modeling, and design [19]. Figure 1 shows that, in contrast to 3D printing, the output product of 4D printing is an active or dynamic structure that can be activated with appropriate external stimulus or energy input. The development of the new 4D printing industry is directly dependent on material science and the development of new materials. In addition to the materials growing and advancing in technologies such as fused deposition modeling (FDM) [20] or fused filament fabrication (FFF), digital light processing (DLP) [21], stereolithography (SLA) [22], selective laser melting (SLM), and inkjet [23,24], it is also a condition for further progress in this field. Various materials such as PVC [25,26], PETG [27], and photopolymers [28] are used in 4D printing, and even blends [29] and multimaterials for 3D-printed auxetic structures [30] are used in 4D printing. This variety of materials and printing technologies, and even the creation of composites, opens completely new perspectives and possibilities for the use of 4D printing in various fields.

PLA is a material that has many applications, both in medicine and non-medical fields. One of its key features is its biocompatibility, which makes it safe for use in medical treatments. As it is a product of the human body and is obtained from natural sources, it is also biodegradable. This is especially important for medical applications, where the device needs to be absorbed by the body after it has served its function.

There is a growing trend of replacing devices composed of metal or alloys with polymers to allow for the gradual healing of diseased tissue through the mechanical weakening of the polymer devices. Additionally, as biodegradation occurs over time, there is no need for additional procedures to remove the device [31]. Due to the ability to customize the chemical structure and mechanical characteristics to the biochemical environment, PLA is widely used in biomedicine. It is used in various applications, including stents [32], orthopedic screws [33], supports for growing various cells, muscle tissue, bone and cartilage regeneration, planting osteogenic stem cells and implantation into bone defects [34], and drug delivery and delivery devices [35]. The use of PLA in additive manufacturing enables the production of complex biomedical devices based on computer-aided design and construction (CAD); in particular, with the use of patient-specific anatomical data, it leads to the creation of one-of-a-kind implants [36] and prosthesis sockets [37]. A new challenge in the field of additive technologies is the application of 3D printing in the production of PLA composites, with or without reinforcement [38], scaffolds [39], biodegradable stents [40] and, lately, in auxetic energy absorption structures [41,42,43,44,45,46,47]. PLA can also be blended with other materials such as TPU in order to show that, by changing the composition and programming temperature, the desired properties for different applications can be achieved so that the highest fixity, recovery, and stress recovery are obtained in hot-, cold-, and warm-programmed samples by manipulating the input energy and temperature [48]. Besides other thermoplastics used in FFF, PLA also shows potential for blending with natural materials such as wood [49].

In the last decade, the number of papers with mechanical tests of FFF samples has increased. In ref. [50], the authors compared the mechanical characteristics of the unidirectional 3D-printed material with that of homogeneous injection-molded PLA, showing that manufacturing by 3D printing and annealing improves the toughness of samples. One of the latest research studies dealt with the influence of strain rate and temperature on the mechanical behavior of a PLA printed structure in tension [51]. The study aimed to analyze the effect of the infill line distance of 3D-printed circular samples on their compressive elastic behavior during cyclic compressive loading [52]. In the paper presented in [53], uniaxial tensile responses of 3D-printed polylactic acid (PLA) samples following standard ASTM-D412 have been studied to characterize the mechanical properties at three temperatures: 30 °C, 40 °C, and 50 °C. Also, this study includes quasi-static compressive experiments performed on polymetric tubes with different temperatures. In ref. [54], the authors conducted experimental testing to determine the compression performance and deformation behavior of 3D-printed PLA lattice structures.

In order to determine the influence of anisotropy and infill on the SME effect in printed materials, the authors in [55] examined the samples using uniaxial tensile tests and compressive tests to study the effect of infill patterns on mechanical properties. Paper [56] presents an experimental study on the compression of uniaxial properties of a PLA material manufactured with FFF in accordance with the requirements and conditions established in the ISO 604 standard, characterizing the compression stiffness, the compression yield stress, the field of displacements, and stress along its elastic area until it reaches the compression yield stress and ultimate yield stress data; the results showed that PLA material is promising for the manufacture of low-volume industrial components that are subject to compression.

The authors in [57] introduced a novel honeycomb structure that can enhance the compression property and energy absorption 4D printing with PLA materials, showing that the novel honeycomb had a high compression property and had high energy absorption capacity. In this work [58], the influence of several factors such as printing temperature, bed temperature, printing speed, fan speed, and flow was studied, showing that the parameters of extrusion-based 3D printing influence the transformability of PLA-based materials. In ref. [59], PLA was used in the 4D printing process for the manufacturing of complex geometry absorber components produced by FFF with varying printing parameters (temperature at the nozzle, the deposition speed, the layer thickness) and activation temperatures. The experiments showed that the components had good shape memory properties that were mostly influenced by activation temperature. Experimental tensile and compression tests were conducted in [60] on FFF PLA parts to evaluate the difference of main mechanical properties in the tensile and compressive state.

In paper [61], the monotonic, fatigue, and creep behavior of PLA under compression was studied using cylindrical specimens that were tested according to ASTM D695 to identify and quantify the effects of printing parameters on the compression behavior of these specimens and failure mechanisms, finding that compressive strength is linearly dependent with the density of the samples. In paper [62], the authors examined PLA and PLA-Cu samples under both static and dynamic loading using a universal testing machine and a split Hopkinson pressure bar apparatus, showing that the addition of copper powder increased the yield strength of the composite material significantly compared with pure PLA, with both materials being strain rate-sensitive. Also, study [63] examined the strain rate sensitivity of five thermoplastic materials (PLA, ABS, PC, CPE+, and nylon) under various tensile test speeds to study strain rate influence on the mechanical characteristics of FFF 3D-printed materials. The influence of strain rate on tensile strength and yield strength in dynamic conditions was examined.

The compression behavior of 4D-printed metamaterials with various Poisson ratios in [64] showed that cellular metamaterials with zero Poisson ratios possessed superior vibration isolation capability compared with negative or positive Poisson ratio cellular metamaterials at different deformation stages by using a comprehensive analysis. A very detailed study presented in [65] described the influence of printing parameters on the mechanical response of polylactic acid (PLA), high-impact polystyrene (HIPS), and acrylonitrile–butadiene–styrene (ABS), with special reference to shape memory in a 4D print while stretching at different speeds and at different temperatures. In order to examine the tensile strain rate performance of 3D-printed PLA with various printing orientations in paper, ref. [66] conducted a study using different strain rates ranging from the slowest to medium speed. The study, like most of the previous ones, showed different responses when the rate of deformation increased using an additional analysis of elongation and bending.

Even though the thermo-mechanical behavior of 4D-printed PLA has been studied for years, comprehensive stress–strain data regarding a strain of ≈50%, including loading and unloading, a variety of strain rates in the range of 0.0001 to 0.1 s^−1^, and temperature ranges of 23 to 50 °C are not available. This paper aims to extensively and experimentally investigate the dependence of FDM 4D-printed PLA on a wide range of strain rates and temperatures in compression scenarios with large deformations. Due to high strain rates, test conditions can occur that are almost adiabatic. Determining the existence of self-heating in FDM PLA and the consequent additional softening is a special challenge. All tests were carried out in coupled thermo-mechanical conditions so that the research results contribute to the expansion of knowledge in the field and provide new insights into the behavior of 4D-printed PLA. The main motive is to determine all parameters related to the macro-mechanical characteristics of the material, which will assist the development of a coupled thermo-mechanical constitutive model for accurately modeling the behavior of the material using the finite element method (FEM). The most important motive is the possibility of expanding knowledge and further research in the field of auxetic structures, whose primary mode of use and exploitation is radial and uniaxial compression at various strain rates.

In the Section 2, a comprehensive outline of the materials and methods utilized is presented, encompassing details such as the filament used for the 3D printing of samples, sample annealing procedure, uniaxial isothermal tensile filament, compression of cylindrical printed samples, and DMTA; the findings obtained through DMTA analysis and the uniaxial tensile and compression testing are eloquently presented and thoroughly discussed within the context of the paper’s primary objectives. The paper is concluded with a summary of the most critical findings and thoughtful suggestions for future research.

## 2. Materials and Methods

### 2.1. Three-Dimensional Printing of PLA Samples

**Figure 2:**
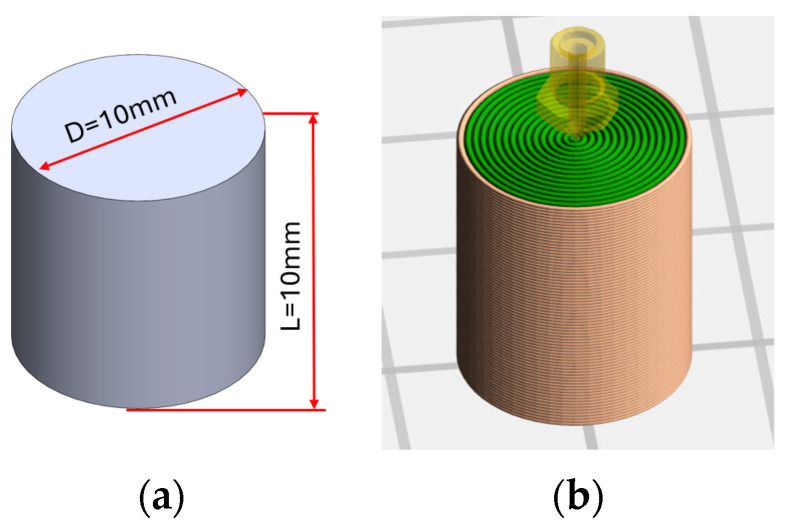
(**a**) geometry of 4D-printed sample and (**b**) 3D printing preview of slicing patterns.

The required PLA samples for all thermo-mechanical uniaxial compression tests were printed by 3D printing a PLA filament with a diameter of 1.75 mm, manufactured by Ultimaker (Utrecht, The Netherlands). Based on the data sheet of the manufacturer, the material has a density of 1.24 $\frac{g}{cm^{3}}$, a melting rate during printing (MFR) of 6.09 $\frac{g}{10\;min}$, and a melting temperature of 145–160 °C. The samples used in this study were manufactured using a UM2 + FDM 3D printer (Ultimaker, Utrecht, The Netherlands) equipped with a 0.4 mm nozzle. The 3D printing settings were chosen based on the manufacturer’s recommendation and experience with a 100% infill, nozzle temperature of 210 °C, working plate temperature of 60 °C, layer deposition height of 0.1 mm, and printing speed of 40 $\frac{mm}{s}$. Cura 5.3.0 (Ultimaker, Utrecht, The Netherlands) slicer software was used for preparing the G-code for the 3D printer. Barreling and buckling of the samples were avoided with the model’s orientation as observed in previous studies, where it shown that these phenomena can be avoided at a ratio of height and diameter of below 2 [67,68,69]. The geometry of the samples and the later test procedure have been defined according to the standard for compression tests ASTM D695 [70], as seen in Figure 2. All tests were performed to obtain stress–strain curves at the maximum safe strain value, e.g., before crack or fracture initiated in the samples. It should be noted that although one of the main advantages of additive manufacturing is the production speed, when considering smart materials in additive technologies, especially within 4D printing, the printing speed must be significantly reduced. As shown in [71], the production of samples in 4D printing at high printing speeds causes extremely high anisotropy due to residual thermal deformations. The basic parameters used in FDM printing for these samples are outlined in Table 1.

### 2.2. Dynamic Mechanical Thermal Analysis (DMTA)

**Figure 3:**
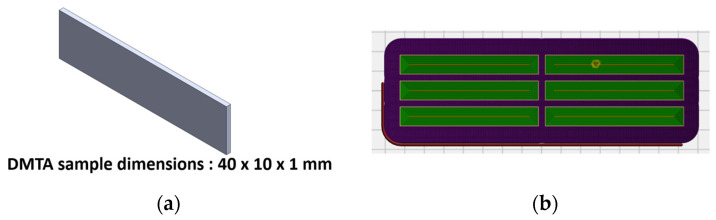
(**a**) Geometry of DMTA samples and (**b**) 3D printing preview of slicing patterns.

The dynamic viscoelastic properties and thermal behavior of 4D-printed PLA were investigated in this study. The experiments were conducted on a solid clamping tool for measurements according to DIN/ISO 6721-1 with the use of Thermo Scientific™ HAAKE™ MARS™ Rheometers (Thermo Fisher Scientific Inc., Waltham, MA, USA) in combination with a controlled test chamber (CTC). The measurements were taken over a temperature range of 40–80 °C, with a strain amplitude of 0.01% and deformation frequency of 1 Hz on standard test prismatic PLA samples; their dimensions were 40 × 10 × 1 mm, and they were fabricated using the same printing parameters that are given in Table 1. The heating rate was set to 2 °C/min, and samples were preheated from room temperature to 40 °C and then kept for 5 min at that temperature. This controlled setup adhered to the guidelines outlined in DIN/ISO 6721-1, ensuring accurate determination of the dynamic mechanical properties of the PLA material under investigation. A DMTA was performed in torsion with a rotational rheometer, where the material was subjected to oscillatory shear while undergoing continuous temperature variation. The geometry of DMTA test samples and the 3D printing preview are shown in Figure 3.

### 2.3. Annealing Printed Specimen

After production, the samples were annealed in an oven at a temperature that was ≈20 °C higher than T_g_ and kept at that temperature for two hours. After that, the samples were slowly cooled in the oven to room temperature over several hours. The annealing procedure after sample fabrication provides several improvements, including reducing imperfections in the samples, reducing porosity, causing better adhesion of material layers, and improving the quality of surface layers. All samples used in the experiments were left in the oven environment (room temperature and humidity of 50%) to eliminate possible external influences and material aging before testing.

### 2.4. Uniaxial Tests

**Figure 4:**
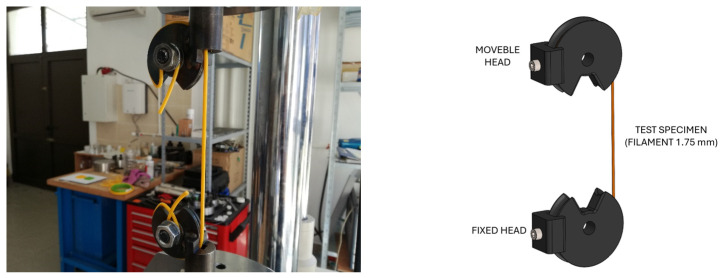
Uniaxial tensile testing of filament procedure: (**left**) equipment for uniaxial filament testing and (**right**) sketch of equipment.

In order to determine the large deformation behavior of 4D-printed PLA polymer at various strain rates and temperatures lower then T_g_, a Shimadzu EHF-EV101K3-070-0A (Kyoto, Japan) universal testing machine equipped with a 100 kN calibrated load cell and temperature chamber was used. Displacement control during the test was performed via the RS485 controller (Schneider Electric, Regensburg, Germany), while the temperature in the temperature chamber was controlled via the EUROTHERM 2408 controller (Worthing, West Sussex, UK) and the iTOOLS 9.87 software package. As the diameter of the filament at 1.75 mm was not adapted to the tension grips, the adaptive tool shown in Figure 4 was used during testing. Tensile tests were performed at room temperature for three strain rates, 0.01, 0.001, and 0.0001 s^−1^.

Thermo-mechanical properties in uniaxial compression were measured using a Shimadzu EHF-EV101K3-070-0A universal testing machine (Kyoto, Japan) equipped with a 100 kN calibrated load cell and temperature chamber. Thermocouples were placed close to the surface and at the height of the sample, while the temperature of the chamber in the sample zone was maintained. Cylindrical samples with a height and diameter of 10 mm were used; the ratio of height and diameter was chosen in order to avoid the occurrence of barreling and buckling of the samples as observed in previous studies, where it was shown that these phenomena occur at a ratio of height and diameter exceeding 2. To reduce the friction, Teflon strips were placed between the sample and the surface of the compression platens. The procedure of isothermal tests was defined as follows: the specimen was placed on a previously applied Teflon (PTFE) strip on the bottom compression platen; then the chamber was heated to the desired test temperature. In order to achieve temperature equilibrium, the specimen was kept in the heated chamber ≈30 min before starting the uniaxial compression test. The upper moving platen moved freely for a given displacement at a constant strain rate for the compression tests. All successful experiments were repeated three times for each strain rate and temperature (36 specimens in total) to ensure the repeatability of results and exclude potential mistakes. Uniaxial compression experiments were conducted at four different strain rates, 0.1, 0.01, 0.001, and 0.0001 s^−1^, and at three temperatures, 25, 37, and 50 °C. The mechanical test results, including the upper and lower limits of measured stresses, can be found in Supplementary Material Figure S1.

### 2.5. Measurement of Temperature Change at High Strain Rates

This study examined PLA material with a shape memory effect and 4D printing properties. The measurement of temperature increase in the samples was performed at room temperature and at strain rates of 0.01 s^−1^ and 0.1 s^−1^. Even before the initial tests, these strain rates were identified as those for which an almost adiabatic scenario is established for thermoplastics [72,73,74,75]. A Flir I7 infrared camera was used for the measurement of temperature evolution during compression tests.

## 3. Results and Discussion

### 3.1. DMTA

**Figure 5:**
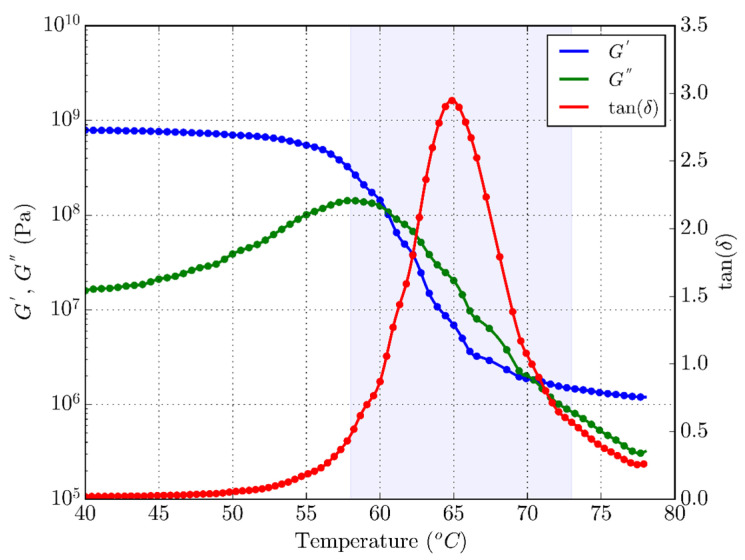
DMTA results for 4D-printed PLA.

Figure 5 allows for the identification of three characteristic zones of PLA: glass transition (blue area in the figure), a solid or glassy phase with high values of elastic moduli (left of the glass transition zone), and a rubbery or soft phase of low values of elastic moduli (right of the transition zone). Figure 5 shows changes in the storage modulus and tan delta in the DMTA results. According to Figure 5, the glass-to-rubber transition zone for PLA starts at 57 °C and continues to 73 °C. Also, the middle value of the storage modulus is detected at 65 °C, which represents the glass transition temperature. The extreme peak of the storage modulus drop occurs in a narrow temperature range. The glass transition temperature of PLA, whose position is defined by the peak of the loss tangent (tan delta), is approximately at T_g_ = 65 °C, at which a significant drop in the elastic modulus occurs. In the given temperature range, the storage modulus decreased from 1045.7 MPa (glassy phase) to 1.2 MPa (rubber phase), and their high ratio (more than two orders of magnitude) shows the great shape memory potential of 4D-printed PLA [76]. Both the drop in the elastic modulus and peak of the tan delta are clear in the marked blue area of the glass transition zone. The results are in agreement with previous studies [77,78] for 4D-printed PLA, Table 2 shows the summary of the results of measuring the transition temperature T_g_ and the changes in the storage modulus G′, loss modulus G″ and loss tangent tan delta with the temperature obtained by the DMTA tests.

### 3.2. Uniaxial Tensile Tests of Filament

**Figure 6:**
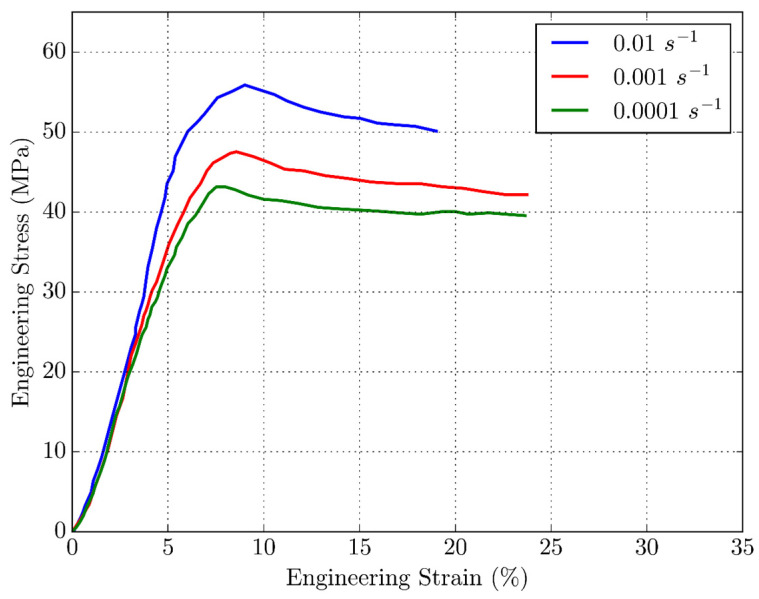
Stress–strain curves for PLA filament at various strain rates.

This section provides confirmation of the dependence of the base material PLA, used for printing the samples, on the strain rate. As expected, Figure 6 shows a typical distribution of curves for thermoplastic materials that depend on the rate of deformation. At all three strain rates, the elastic range up to the point of over-yielding is expressed. The non-linear increase in stress follows up to the yield point, which is also the point of the highest stress, after which deformational softening of the material follows. The yield stresses are 55 MPa, 48 MPa, and 42 MPa for strain rates of 0.01 s^−1^, 0.001 s^−1^, and 0.0001 s^−1^, respectively. Also, all yield points lie in the range of 6–8% of deformation. The results of the tensile filament test are summarized in Table 3.

### 3.3. Uniaxial Compression Testing

**Figure 7:**
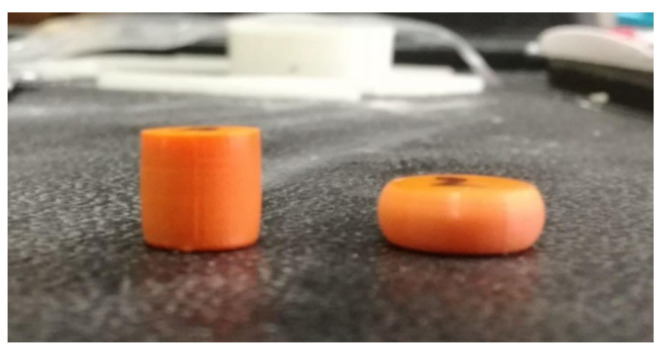
PLA 4D-printed cylindrical samples—as printed (**left**) and after compression (**right**).

A series of uniaxial compression experiments have been conducted on the 4D-printed PLA. All tests were conducted for temperatures below the determined glass transition temperature of T_g_ = 65 °C. The cylindrical compression test samples were 10 mm in diameter and 10 mm tall, created according to previous studies of uniaxial compression. Samples were annealed right after 3D printing by heating in an oven at a temperature about 20 °C above the determined glass transition temperature for two hours before slow cooling to room temperature. The experiments were conducted using a servo-hydraulic Shimadzu testing machine equipped with a thermal chamber. In order to heat the compression steel platens uniformly, sample and steel platens were allowed to heat at the testing temperature for about 30 min prior to testing. To reduce friction at the contact surfaces, Teflon (PTFE) films were applied. Figure 7 shows the as-printed cylindrical samples and samples after the compression test. It can be observed that although friction is present during the test, visual inspection of the edges of the sample shows a reduced effect of friction due to the use of Teflon strips. The uniaxial compression experiments on PLA were conducted for the temperatures of 25, 37, and 50 °C at four strain rates, 0.0001, 0.001, 0.01, and 0.1 s^−1^. The compression tests were conducted at a strain level of ≈50% (0.68 true strain). Because the drop in the mechanical response is observed for the higher strain rates, the analysis of results was separated into two groups: isothermal testing at the strain rates of 0.0001 and 0.001 s^−1^ and adiabatic testing at the strain rates of 0.01 and 0.1 s^−1^.

**Figure 8:**
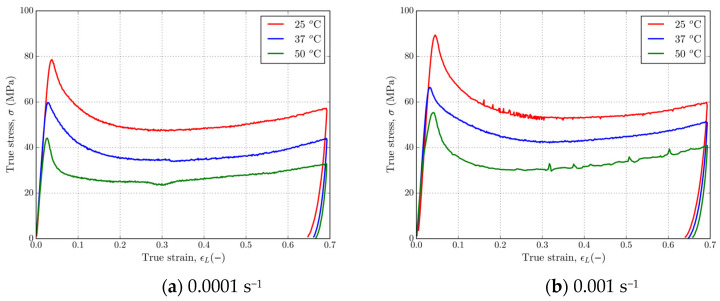
Stress–strain curves in uniaxial compression for PLA at strain rates of (**a**) 0.0001 s^−1^ and (**b**) 0.001 s^−1^ and temperatures of 25, 37, and 50 °C.

**Figure 9:**
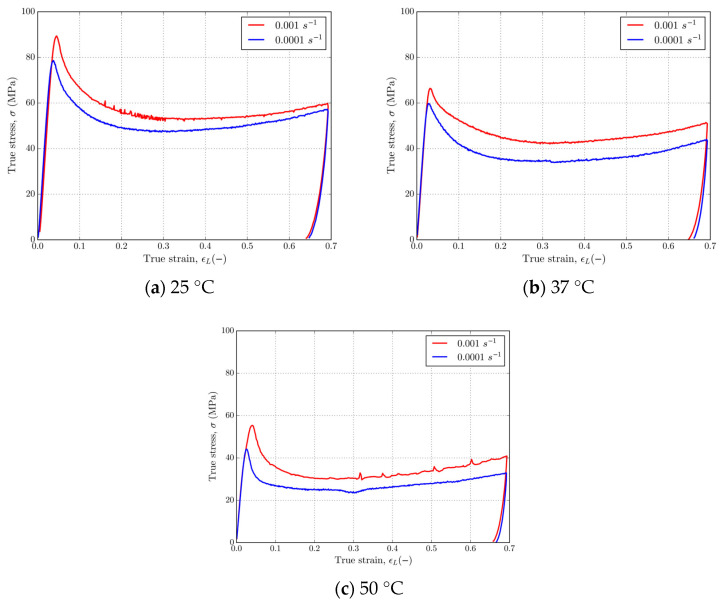
Stress–strain curves in uniaxial compression for PLA at temperatures of (**a**) 25 °C, (**b**) 37 °C, and (**c**) 50 °C at strain rates of 0.0001 and 0.001 s^−1^.

Figure 8 and Figure 9 show that 4D-printed PLA polymer exhibits a strain rate and temperature-dependent response typical for solid or glassy phases under isothermal experimental conditions. The material has shown a tendency of glassy polymer behavior under a T_g_ with an initial elastic region and rate-dependent yield point, followed by strain softening and strain hardening at larger strains. Figure 8 shows representative stress–strain curves for PLA at strain rates of 0.0001 and 0.001 s^−1^ at temperatures of 25, 37, and 50 °C. Referring to Figure 8, it can be observed that as the temperature increases from 25 to 50 °C, the yield stress decreases from ≈80 MPa to ≈40 MPa for the strain rate of 0.0001 s^−1^ and from ≈90 MPa to ≈60 MPa for the strain rate of 0.001 s^−1^. In the case of both strain rates, strain hardening at large strains is present. Figure 9 shows a set of stress–strain curves for the strain rates of 0.0001 and 0.001 s^−1^ at temperatures of 25, 37, and 50 °C. Referring to Figure 9, which shows stress–strain curves at various fixed temperatures and at two different strain rates, a clear strain rate dependence is observed in the material. In this case, the yield stress of the material decreases by ≈10 MPa for each decade decrease in strain rate at given temperatures, resulting in ≈80 MPa and ≈70 MPa, ≈70 MPa and ≈60 MPa, and ≈60 MPa and ≈50 MPa for the temperatures of 25, 37, and 50 °C, respectively. Upon unloading, about a 4% strain is reversible when the temperature is held constant. Increasing the temperature at the same strain rate leads to a drop in stress during strain hardening, but the amount of hardening at larger strains is slightly affected for this temperature range. Even for the very large strains, there is no intersection of stress–strain curves, which indicates that no additional softening caused by the strain rate occurred.

**Figure 10:**
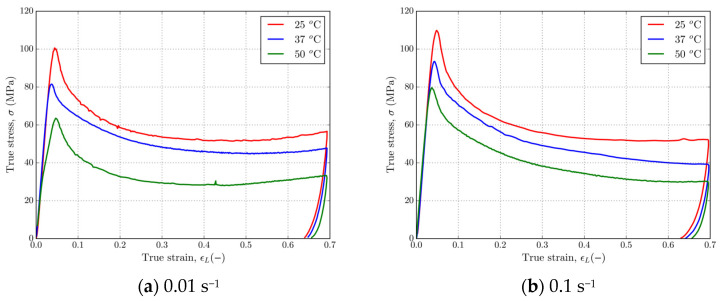
Stress–strain curves in uniaxial compression for PLA at strain rates of (**a**) 0.01 s^−1^ and (**b**) 0.1 s^−1^ and temperatures of 25, 37, and 50 °C.

**Figure 11:**
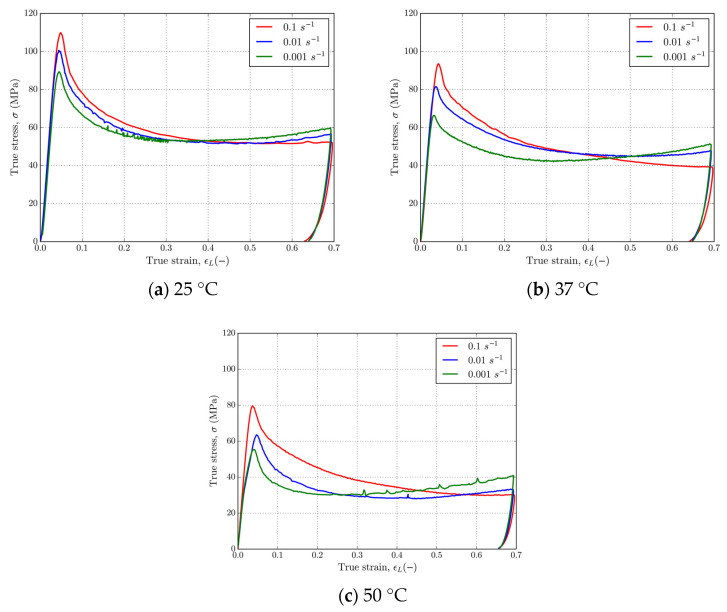
Stress–strain curves for PLA at temperatures of (**a**) 25 °C, (**b**) 37 °C, and (**c**) 50 °C at strain rates of 0.01 and 0.1 s^−1^.

Figure 10 and Figure 11 show that 4D-printed PLA polymer exhibits a strain rate and temperature-dependent response typical for solid or glassy phases at adiabatic experimental conditions. Figure 10 shows representative stress–strain curves for PLA at strain rates of 0.01 and 0.1 s^−1^ at temperatures of 25 °C, 37 °C, and 50 °C. Referring to Figure 10, it can be observed that as temperature increases from 25 to 50 °C, the yield stress decreased from ≈100 MPa to ≈60 MPa for the strain rate of 0.01 s^−1^ and from ≈110 MPa to ≈80 MPa for the strain rate of 0.1 s^−1^. Slight strain hardening at large strains is present only at a strain rate of 0.01 s^−1^. Figure 11 shows a set of stress–strain curves for the strain rates 0.001, 0.01, and 0.1 s^−1^ and temperatures 25 °C, 37 °C, and 50 °C. It should be noted that a 0.001 s^−1^ strain rate curve has been added to this figure in order to show the amount of softening and make a clear distinction between isothermal and adiabatic strain rates. Referring to Figure 11, which shows stress–strain curves at various fixed temperatures and at the three different strain rates, clear strain rate dependence is observed in the material. In these cases, the yield stress of material decreased by ≈10 MPa for each decade decrease in strain rate at given temperatures, resulting in ≈100 MPa, ≈90 MPa, and ≈80 MPa; ≈90 MPa, ≈80 MPa, and ≈70 MPa; and ≈80 MPa, ≈60 MPa, and ≈50 MPa for the temperatures 25 °C, 37 °C, and 50 °C, respectively. In this case, another very important strain rate-dependent feature of PLA is observed for the higher strain rates 0.01 and 0.1 s^−1^ at all temperatures. At higher values of strain, in the case of both higher strain rates, heat generated during plastic deformation could not be dissipated to the surrounding area, which is a clear explanation for the crossing of curves at higher strains, which is in correlation with the tensile [79] and compression [73,74] results of previous studies. Upon unloading, about ≈5% strain is reversible when the temperature is held constant.

The characteristics that can be observed in the stress–strain curves are yield curves that have a clearly defined yield point, like the curves grouped by strain rate, and after unloading, ≈5% of strain is reversible. There is an intersection of the strain–stress curves due to the stress drop at strain rates of 0.1 s^−1^ and 0.01 s^−1^ at deformation ≈0.5, which is in agreement with the results for thermoplastics [75,80,81]. The stress drop is a consequence of self-heating in the case of both strain rates; heat cannot be dissipated from the surrounding area because of the speed of the process, which consequently leads to further softening of the thermo-sensitive material.

**Figure 12:**
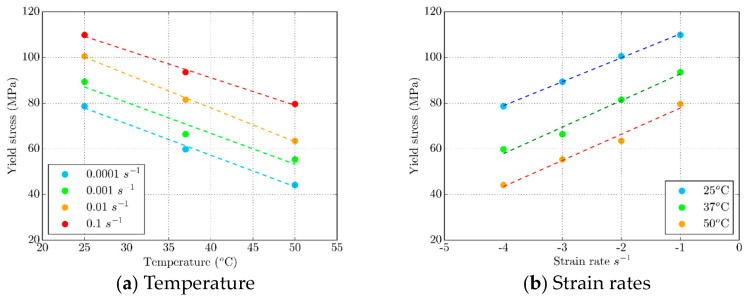
Yield stress value dependence on (**a**) temperature and (**b**) strain rate.

Figure 12 summarizes the dependence of yield stress on temperatures and strain rates. The yield stress lies in a wide range of values, ranging from 110 MPa for the most extreme case of room temperature and the highest strain rate to 42 MPa at a temperature of 50 °C and the lowest applied strain rate. It is important to emphasize that a logarithmic scale was utilized to illustrate the correlation between yield stress and strain rate. This method of organizing the data offers a clearer comprehension of the significance of yield stress in the thermo-mechanical uniaxial compression of PLA. As anticipated, the yield stress decreases as temperatures rise, and strain rates remain constant. On the other hand, increasing the strain rate at constant temperatures leads to a higher yield stress response of the material. These variations are nearly linear across the board, which can be beneficial in establishing the pattern of change when implementing the constitutive model for 4D-printed PLA.

### 3.4. Adiabatic Tests with Self-Heating and Strain Softening

**Figure 13:**
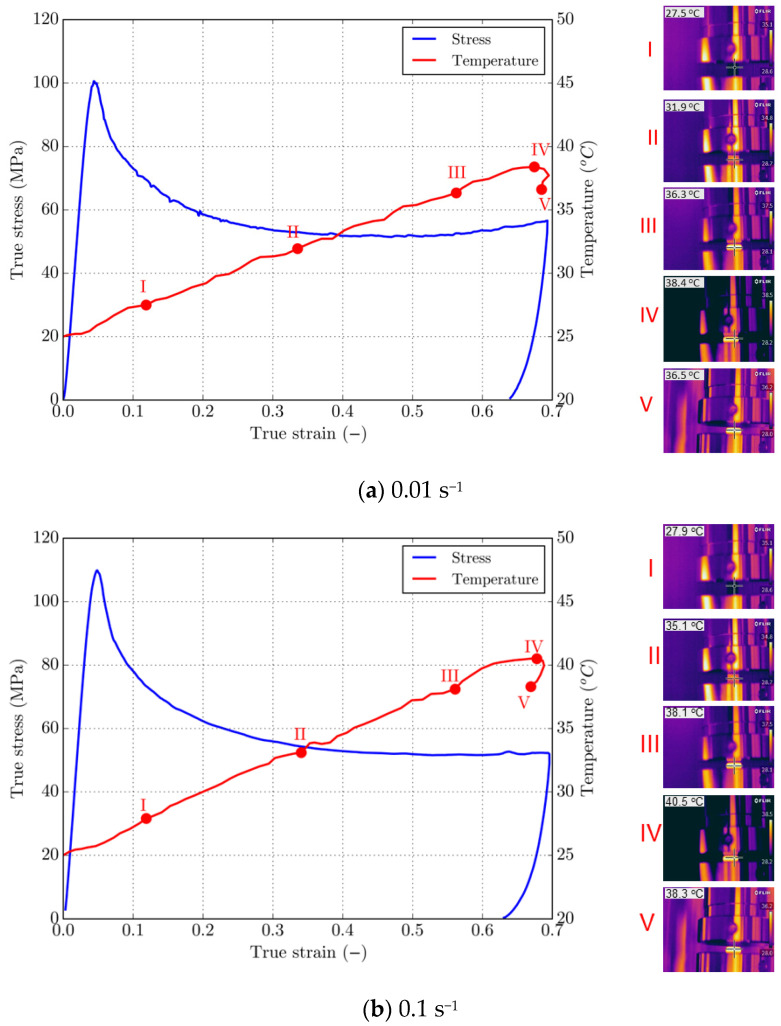
Temperature evolution images in 5 characteristic points (I-V) of 4D-printed PLA at strain rates of (**a**) 0.01 s^−1^ and (**b**) 0.1 s^−1^.

In this section, the results of self-heating in the material at strain rates of greater than 0.01 s^−1^ are presented, indicating that the mentioned processes can be considered almost adiabatic. A typical thermo-mechanical coupled curve is shown in Figure 13. The temperature was recorded with a thermal imaging camera for the PLA sample at strain rates of 0.01 and 0.1 s^−1^. The recording shows that the temperature on the surface of the sample increases monotonously, with the temperature rising from room temperature to ≈40 °C. Thermal imaging reveals an observed temperature increase of 15 °C and 18 °C on the sample’s surface for the strain rates of 0.01 s^−1^ and 0.1 s^−1^, respectively. Although this temperature is below the determined Tg for PLA, as shown in the stress–strain curves, there is a drop in mechanical characteristics that can be attributed to the internal heating of the extremely thermo-sensitive material. The maximum temperature was consistently observed in the middle of the sample. It is also shown that the increase in temperature is insignificant before the flow in material and that the temperature continues to rise constantly. Immediately after the start of unloading, a temperature drop of ≈2 °C is observed. These findings will serve as the foundation for upcoming research, which will center on employing advanced material modeling methods, including thermo-mechanical coupling, to address self-heating effects.

## 4. Conclusions

This study confirmed PLA material’s dependency on strain rate, with the stress–strain curves displaying typical thermoplastic behavior. Yield stresses varied with strain rates, underscoring the material’s sensitivity to strain rates. The samples exhibited clear strain rate dependence, particularly at quasi-static rates, with temperature and strain rate variations significantly impacting mechanical properties, including yield stress and deformation behavior. Isothermal compression tests showed predictable stress–strain curves with distinct yield points, while adiabatic tests revealed additional complexities, such as heat accumulation leading to further softening. Observations at higher strain rates indicated self-heating phenomena in PLA, resembling adiabatic conditions. Thermal imaging revealed temperature increases during deformation, with maximum temperatures occurring at the sample’s center. The drop in mechanical characteristics attributed to internal heating highlighted the material’s thermo-sensitive nature. These findings deepen the understanding of PLA behavior and hold significant implications for practical applications, especially in 3D and 4D printing and manufacturing. Future research should focus on advanced modeling techniques to predict material behavior and explore mitigation strategies for self-heating effects, enhancing PLA-based product reliability and performance in applications with deformations at higher strain rates. The observed adiabatic processes that take place in the material during deformation at high strain rates require the development of an FEM coupled thermo-mechanical constitutive model to simulate self-heating processes with sufficient accuracy. In addition, the ultimate goal of following research is to expand the FEM model with the ability to simulate shape recovery in 4D-printed PLA samples and structures, both at cold (temperatures below T_g_) and hot programming (temperatures over T_g_). Cold programming is essential because most auxetic structures and metamaterials undergo deformation at temperatures lower than T_g_, and the simulation of shape recovery occurs by heating above T_g_. The 4D-printed PLA’s remarkable stability at lower temperatures and ability to undergo significant deformations at higher temperatures make it an ideal candidate for shape recovery research, particularly in auxetics. Its capacity for precise shape retention and adaptive behavior offers innovative applications in biomedical devices, aerospace, and soft robotics, where dynamic responses to external stimuli, like temperature changes, are essential. These findings will serve as the foundation for upcoming research, which will center on employing advanced material modeling methods, including thermo-mechanical coupling, to address self-heating effects. The goal is to improve the reliability and performance of PLA-based products in applications at higher strain rates, especially auxetic and metamaterial structures, and to create models for simulating shape recovery in 4D-printed PLA structures at cold and hot programming temperatures. A study set up this way could be the basis for the successful and precise modeling of auxetics and metamaterials in cold and hot programming in consecutive research. The lower temperatures used in this research should serve to further focus on cold programming auxetics and research related to shape recovery by heating. Although the printing speeds and directions can also affect 4D printing properties, this study focused on a fully thermo-mechanical coupled characterization of PLA to determine characteristics for further developing the constitutive model.

## Figures and Tables

**Table 1:** Printing parameters of testing samples.

Parameter	Value
Nozzle diameter	0.4 mm
Layer height	0.1 mm
Infill	100%
Printing speed	40 mm/s
Printing bed temperature	60 °C
Production time	35 min

**Table 2:** DMTA results.

G′ (MPa)	G″ (MPa)	G′/G″ (−)	T_g_ (°C)
1045	1.2	>100	65

**Table 3:** Results of the filament tensile test.

Strain Rate	Temperature (°C)	Tensile Strength (MPa)	Elongation at Break (%)
0.01 s^−1^	23	55.83 ± 1.54	21.83 ± 3.66
0.001 s^−1^	23	47.83 ± 1.31	30 ± 4.9
0.0001 s^−1^	23	43 ± 0.816	41.33 ± 3.09

## Data Availability

The original contributions presented in the study are included in the article and Supplementary Material, further inquiries can be directed to the corresponding author.

## Supplementary Materials

The following supporting information can be downloaded at: https://www.mdpi.com/article/10.3390/polym16111526/s1, Figure S1: True stress—true strain diagram with upper and lower limits of stresses for all strain rates and temperatures.
