# Supplementary material for: Thermo-Mechanical Behavior and Strain Rate Sensitivity of 3D-Printed Polylactic Acid (PLA) below Glass Transition Temperature (Tg)
Source: Polymers (Basel). 2024 May 29;16(11):1526. doi: 10.3390/polym16111526 (PMC11174730; doi:10.3390/polym16111526)
Supplement: Supplementary file 1 [file polymers-16-01526-s001.zip › polymers-2993978-supplementary.pdf]

Supplementay material 1. Stress-strain curves for all temperatures and strain rates

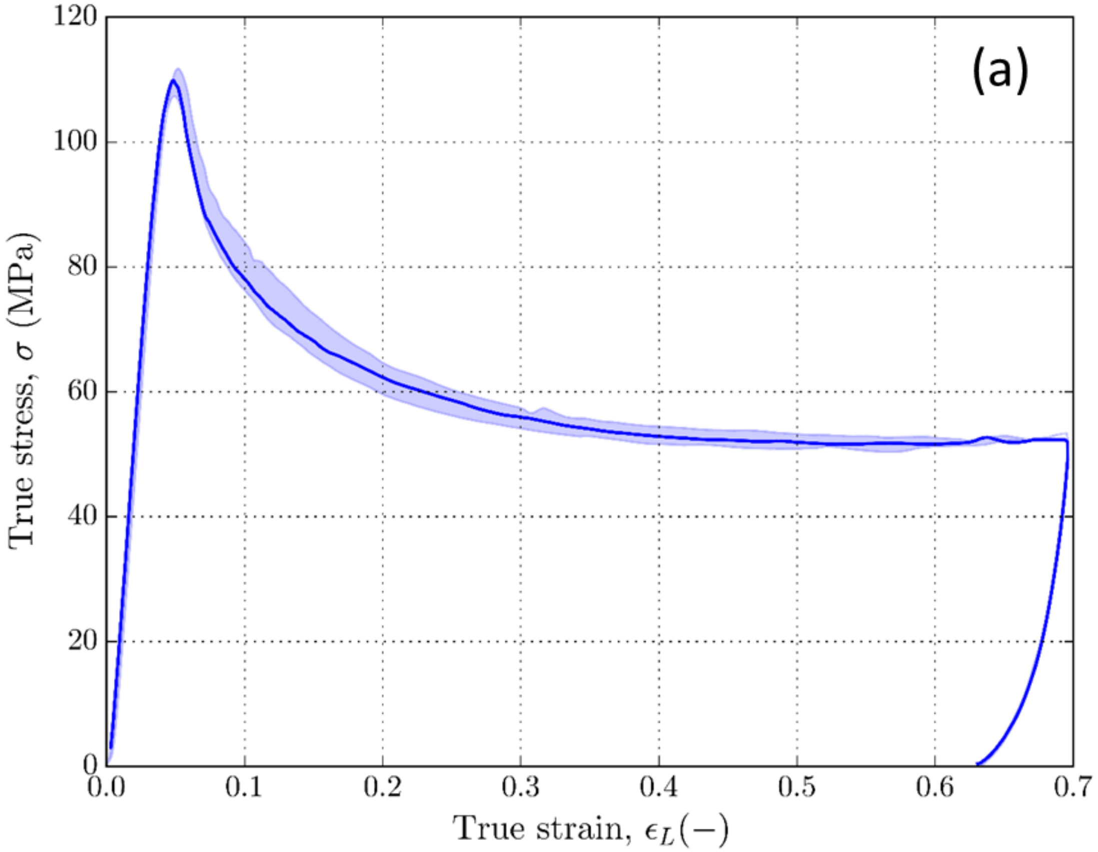

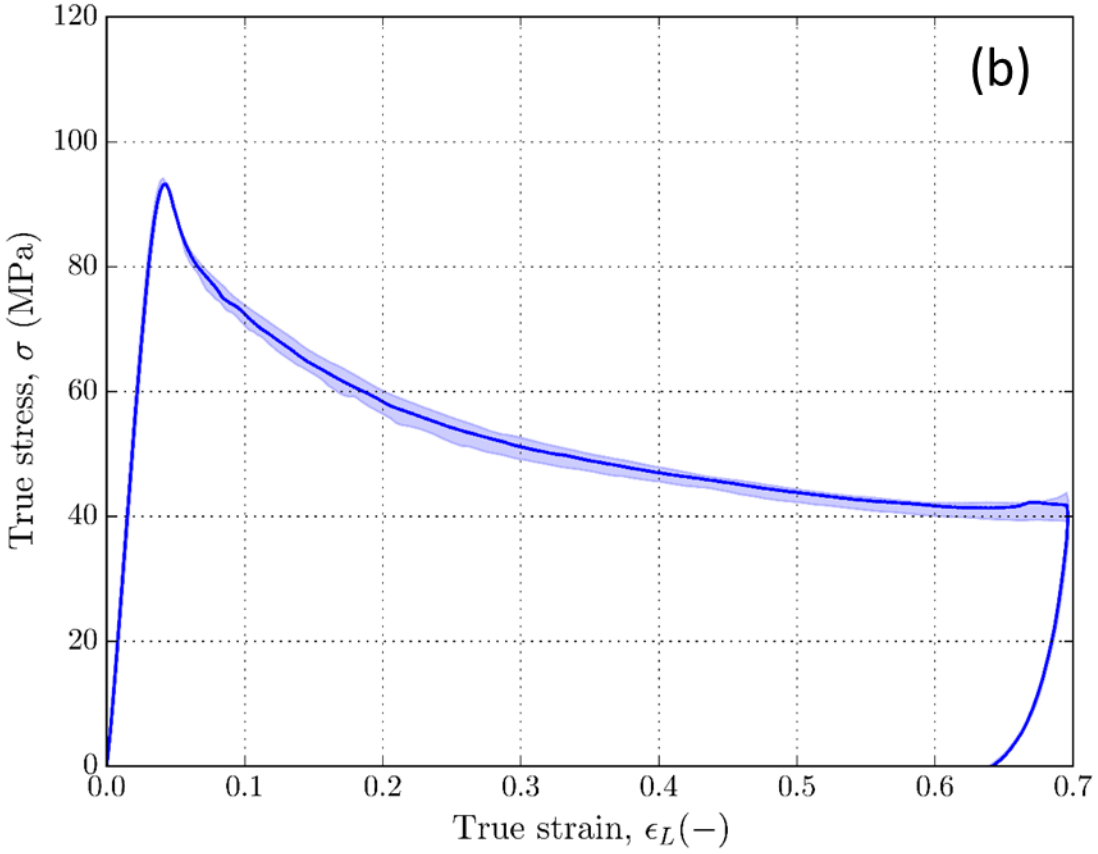

(b)
True stress, σ (MPa)
True strain, $\epsilon_L(-)$
0
20
40
60
80
100
120
0.0
0.1
0.2
0.3
0.4
0.5
0.6
0.7

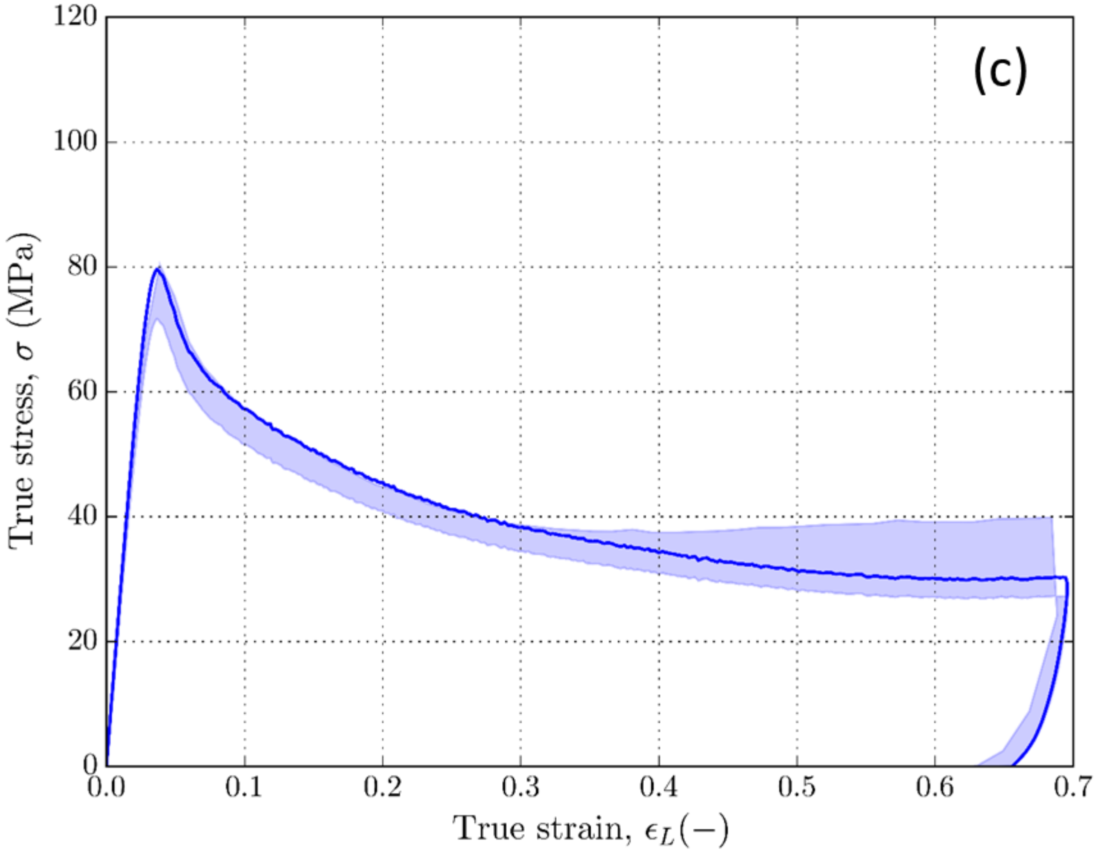

(c)
True stress, σ (MPa)
True strain, ϵ_L(−)
0
20
40
60
80
100
120
0.0
0.1
0.2
0.3
0.4
0.5
0.6
0.7

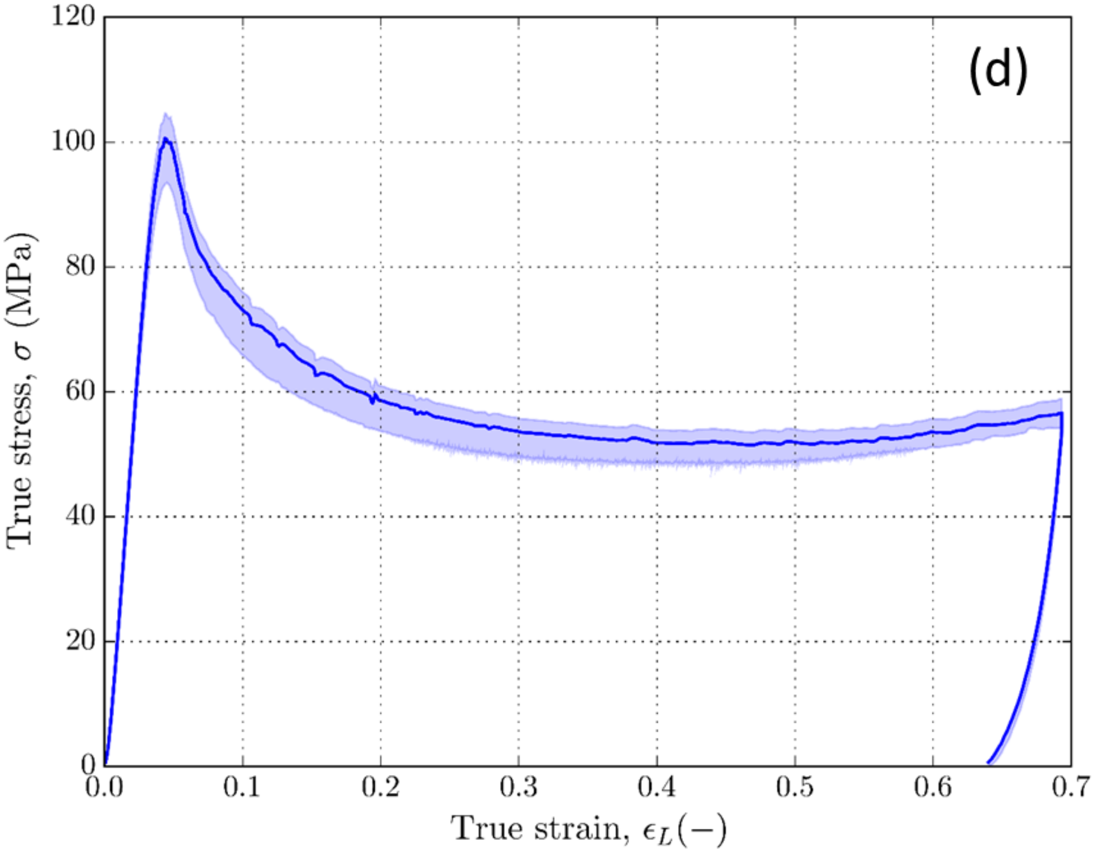

(d)
True stress, σ (MPa)
True strain, $\epsilon_L(-)$
0
20
40
60
80
100
120
0.0
0.1
0.2
0.3
0.4
0.5
0.6
0.7

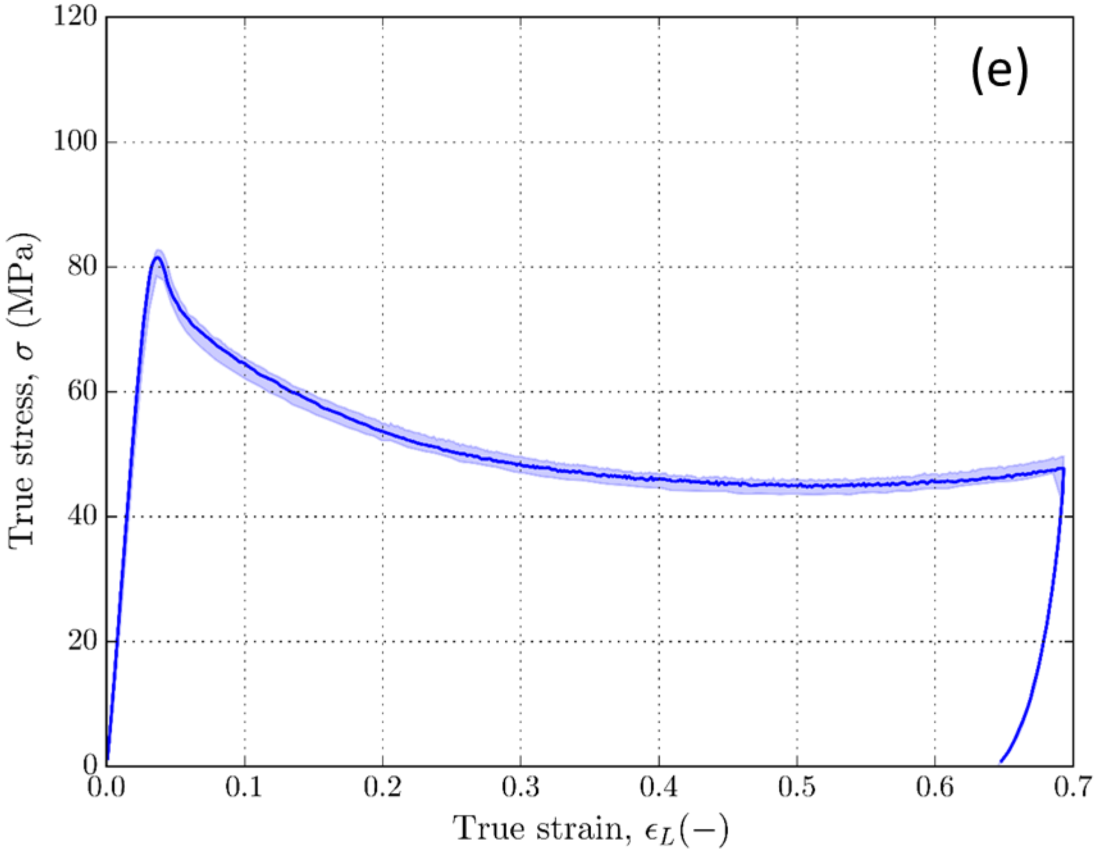

(e)
True stress, σ (MPa)
True strain, $\epsilon_L(-)$
0
20
40
60
80
100
120
0.0
0.1
0.2
0.3
0.4
0.5
0.6
0.7

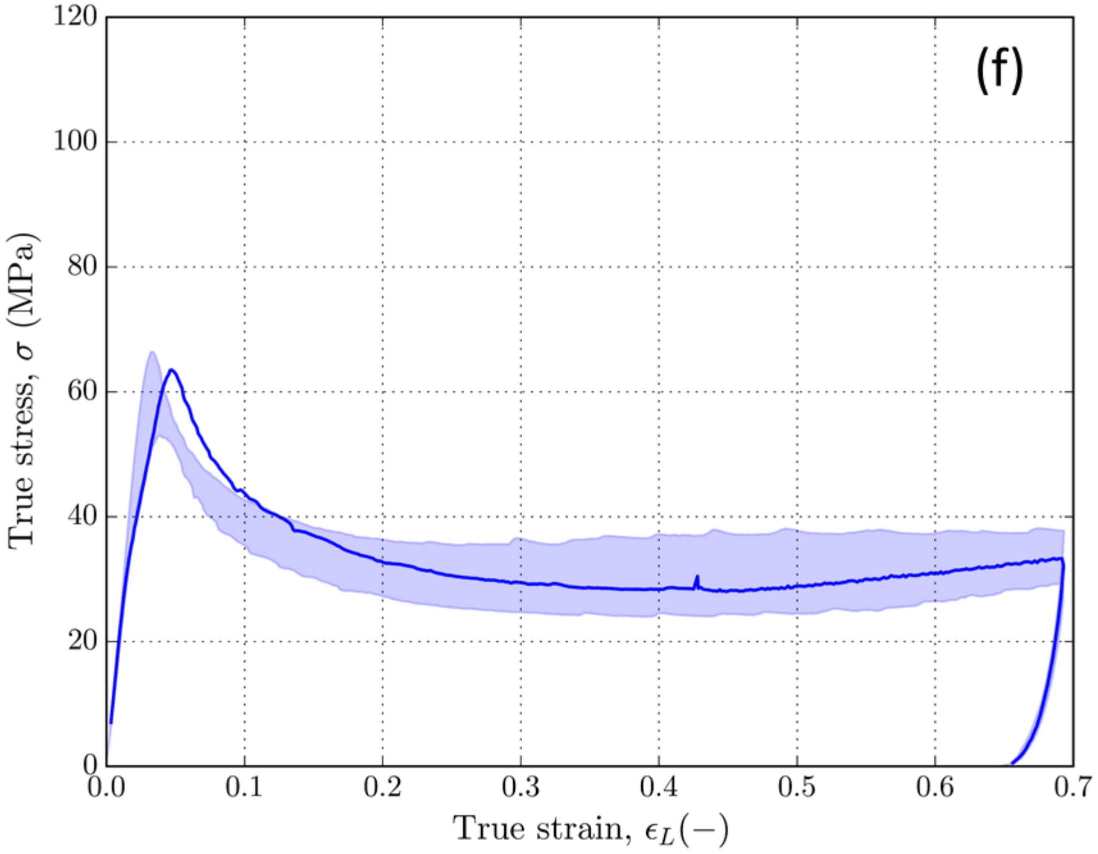
(f)
True stress, σ (MPa)
True strain, $\epsilon_L(-)$
0
20
40
60
80
100
120
0.0
0.1
0.2
0.3
0.4
0.5
0.6
0.7

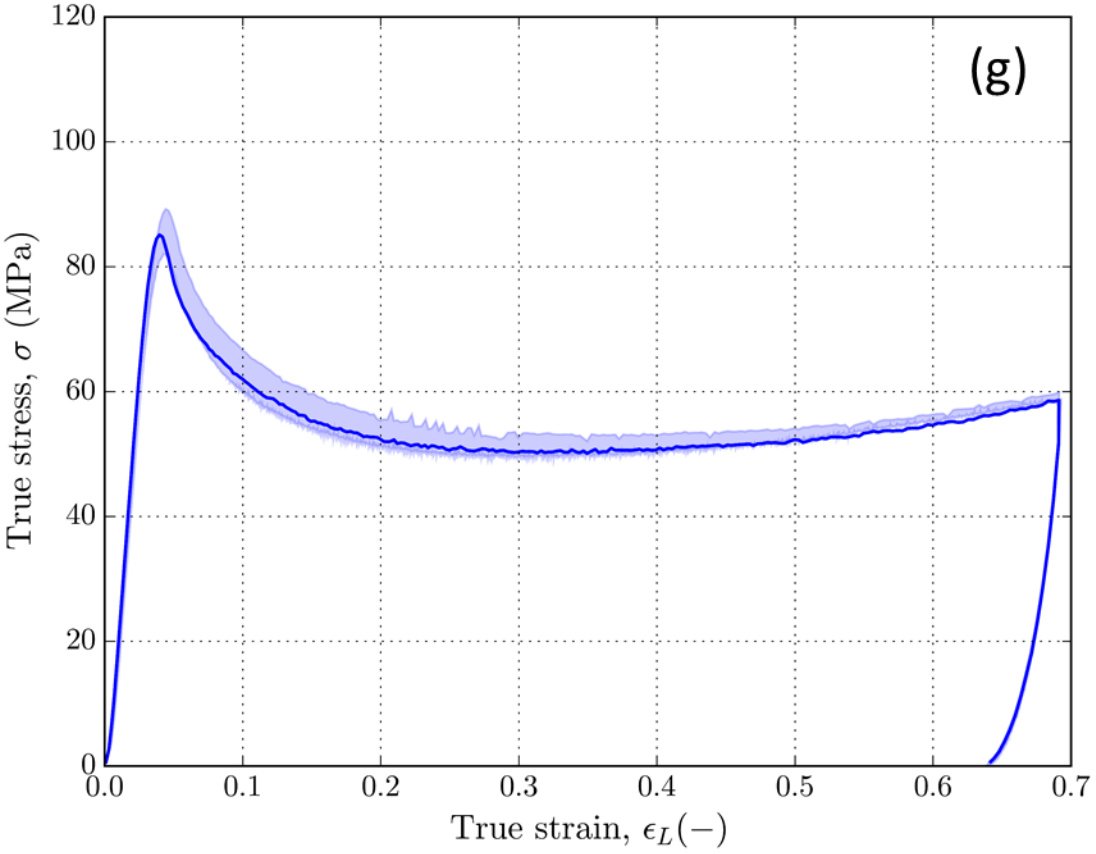
(g)
True stress, σ (MPa)
True strain, $\epsilon_L(-)$
0
20
40
60
80
100
120
0.0
0.1
0.2
0.3
0.4
0.5
0.6
0.7

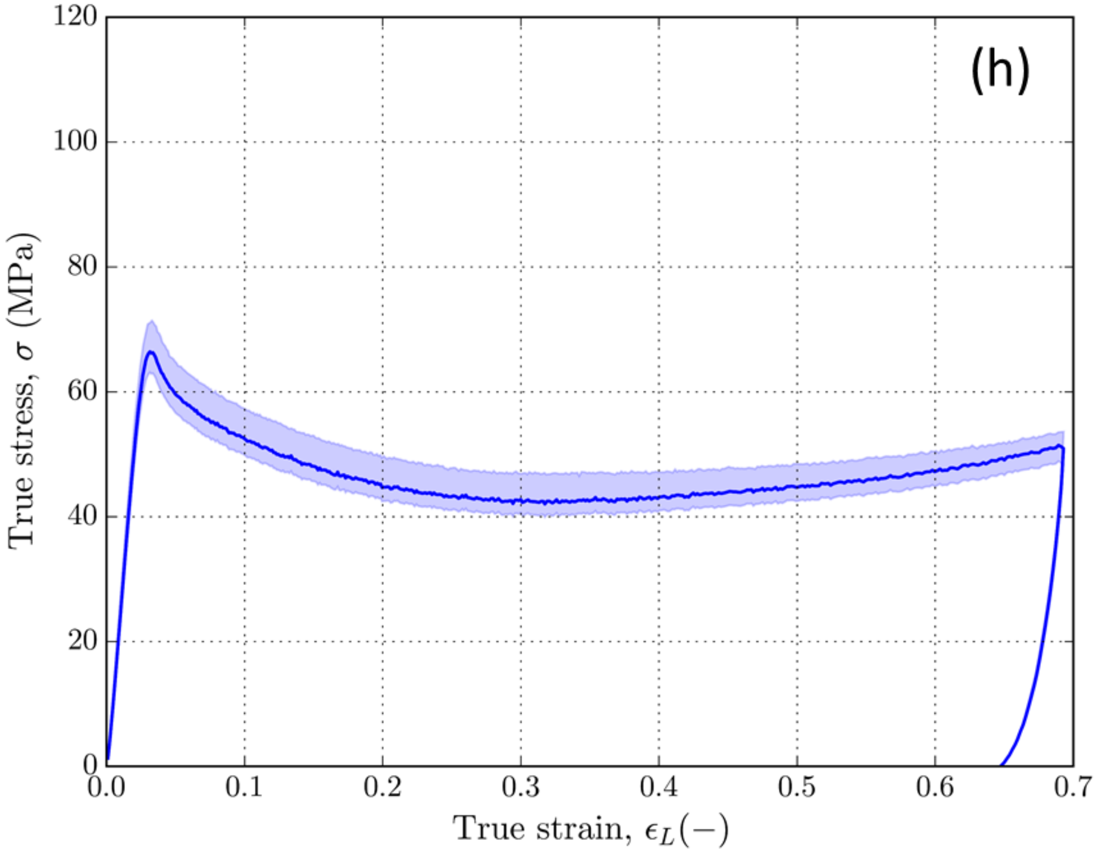

(h)
True stress, σ (MPa)
True strain, $\epsilon_L(-)$
0
20
40
60
80
100
120
0.0
0.1
0.2
0.3
0.4
0.5
0.6
0.7

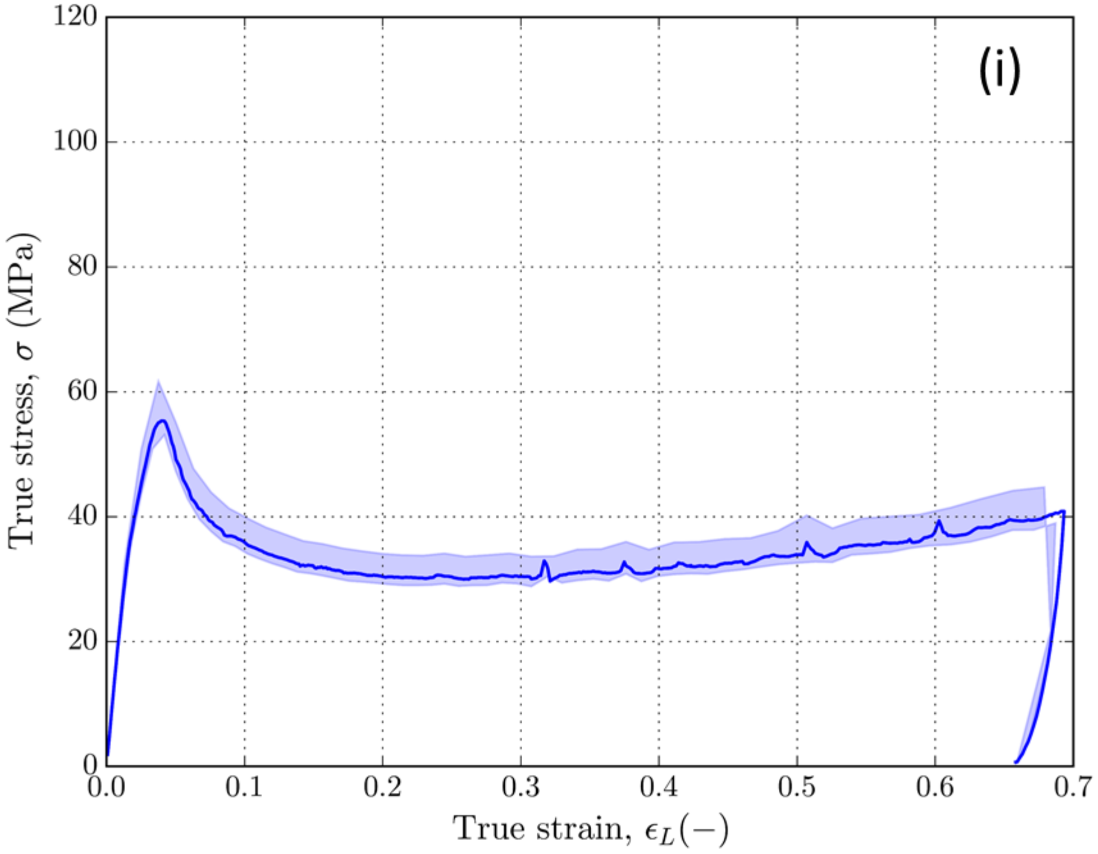
(i)
True stress, σ (MPa)
True strain, $\epsilon_L(-)$
0
20
40
60
80
100
120
0.0
0.1
0.2
0.3
0.4
0.5
0.6
0.7

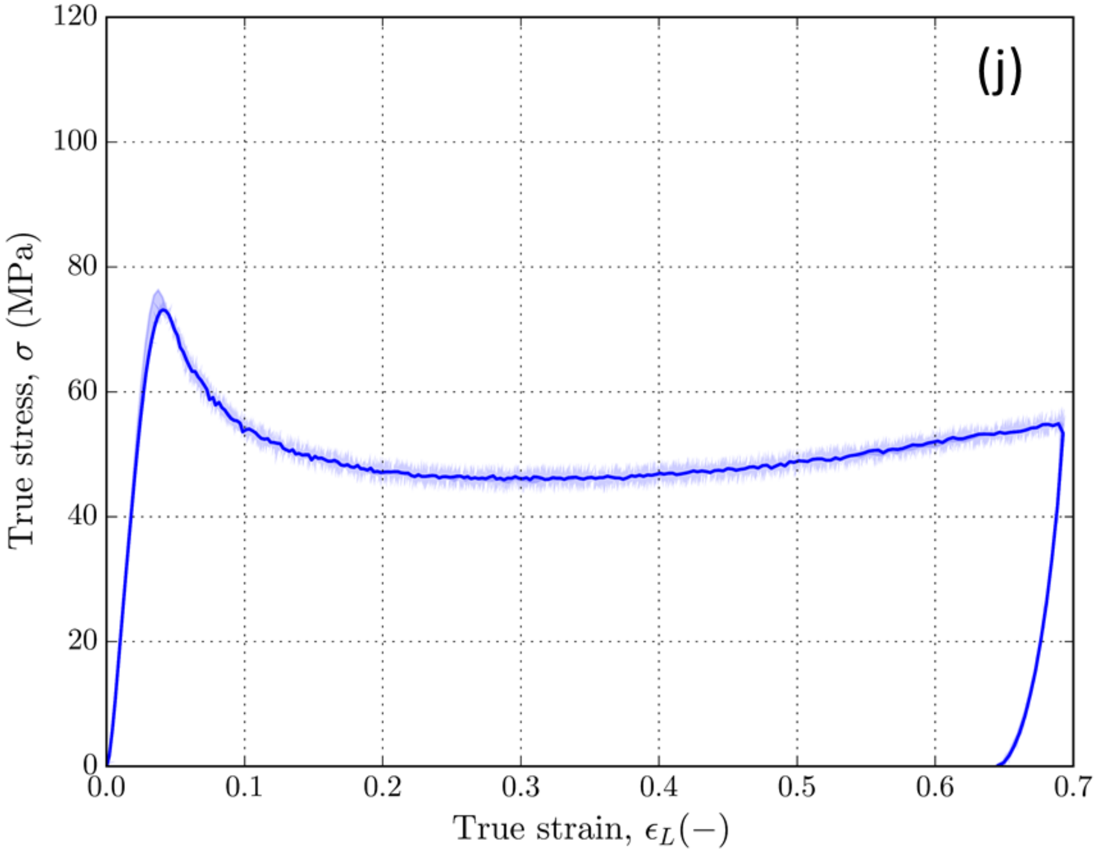

(j)
True stress, σ (MPa)
True strain, $\epsilon_L(-)$
0
20
40
60
80
100
120
0.0
0.1
0.2
0.3
0.4
0.5
0.6
0.7

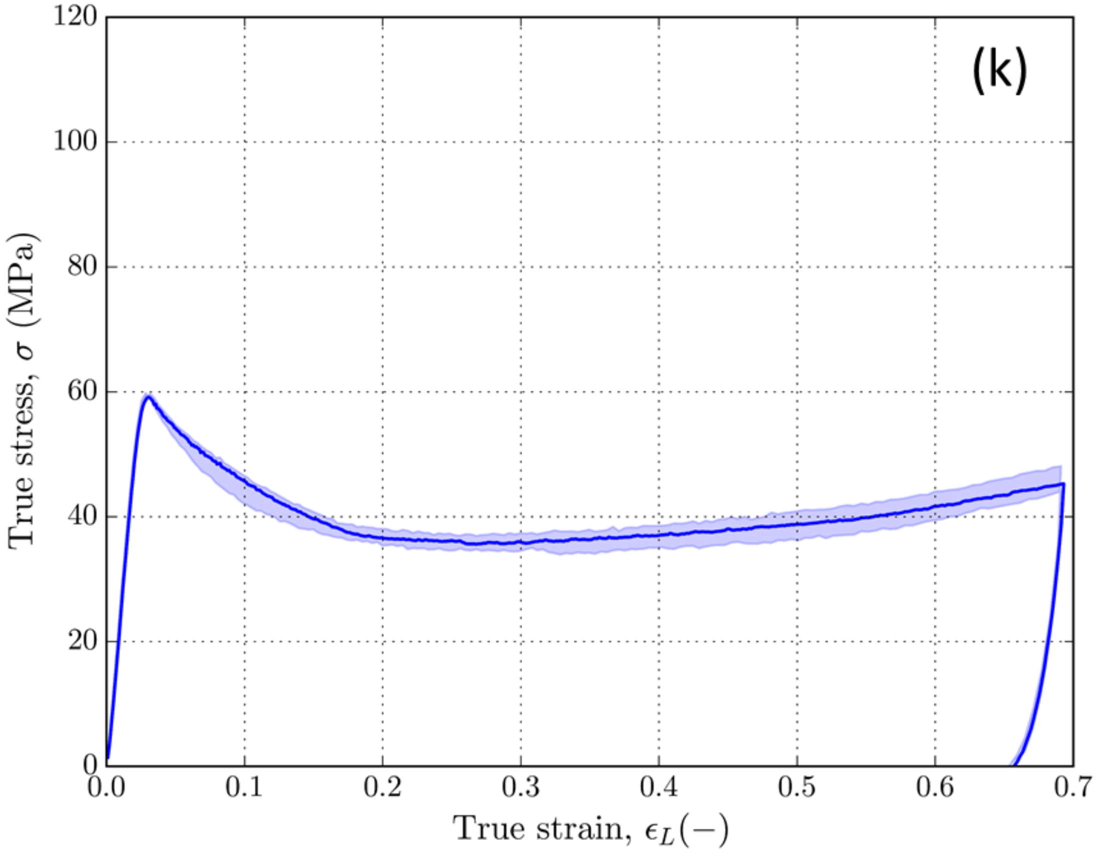

(k)
True stress, σ (MPa)
True strain, ϵ_L(−)
0
20
40
60
80
100
120
0.0
0.1
0.2
0.3
0.4
0.5
0.6
0.7

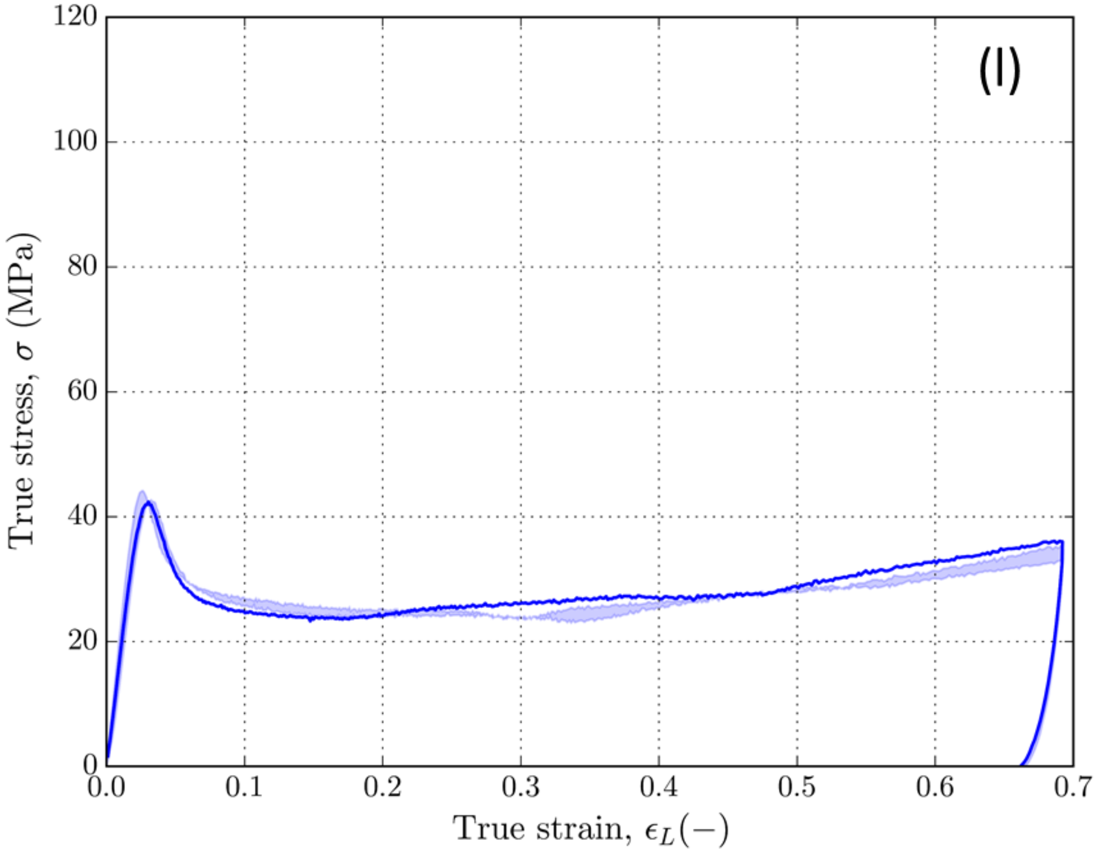

Figure S1. True stress – true strain diagram with upper and lower limits of stresses for (a) 0.1 s^{-1} and temperature 25 °C; (b) 0.1 s^{-1} and temperature 37 °C; (c) 0.1 s^{-1} and temperature 50 °C; (d) 0.01 s^{-1} and temperature 25 °C; (e) 0.01 s^{-1} and temperature 37 °C; (f) for 0.01 s^{-1} and temperature 50 °C; (g) 0.001 s^{-1} and temperature 25 °C; (h) 0.001 s^{-1} and temperature 37 °C; (i) 0.001 s^{-1} and temperature 50 °C; (j) 0.0001 s^{-1} and temperature 25 °C; (k) 0.0001 s^{-1} and temperature 37 °C; (l) 0.0001 s^{-1} and temperature 50 °C
